# The burden is great and the money little: Changing chronic disease management in low– and middle–income countries

**DOI:** 10.7189/jogh.02.020301

**Published:** 2012-12

**Authors:** Daniel D. Reidpath, Pascale Allotey

**Affiliations:** School of Medicine and Health Sciences, Monash University, Sunway Campus, Malaysia

Agenda item 117 of the 66^th^ session of the United Nations General Assembly was a watershed for global health. It marked the adoption by the General Assembly on the 16^th^ of September 2011 of the political declaration of the High–level Meeting of the General Assembly on the Prevention and Control of Non-communicable Diseases [1]. The adoption placed non-communicable diseases (NCDs) center stage for global health. To reach that point required a significant amount of scientific and political effort, first to convene the High–level Meeting on NCDs, and then to have the declaration adopted by the UN General Assembly. The historical time–line leading up to this achievement is punctuated by reflective pieces in a number of journals, but dominated by a series in *The Lancet* [2-8].

One of the interesting features identifiable in the time–line is a shift in vocabulary between late 2010 and early 2011 – a period that is bisected almost exactly by the publication of an article, also in *The Lancet*, identifying “chronicity” as the future issue for health systems [9]. Up until late 2011 the NCDs discussion had more often than not used the vocabulary of chronic diseases rather than NCDs, with reference to a typical set of non-communicable diseases that were chronic in nature, including cardiovascular diseases (mainly heart disease and stroke), some cancers, and type 2 diabetes [2,3]. Occasionally other conditions such as mental health conditions, respiratory conditions, injury and such like would appear in the narrative. The main conditions, however, were those that might be described using a nomenclature of “diseases of lifestyle”, related to choices made about smoking, exercise, and macro– and micro–nutritional content of food [10-13].

The shift in vocabulary may have just been whimsy, but it probably reflected a wish to classify the diseases of interest by their causes rather than by their effects or health systems consequences (long term management) [14]. The global burden of NCDs is significant, and will affect low– and middle–income countries most [15]. As a strategy, there is no doubt that the greatest future health gains in the area of NCDs are going to be made through prevention – which requires an understanding of causation – and might then support the vocabulary shift. Prevention strategies will have to be multifaceted, but may include trying to effect individual behaviour change [16,17], change in industrial behaviour [18] or change in the environment [19]. Making the changes is non-trivial: it will in many cases be harder for lower income countries to implement; it will take time to make the changes; and even when the interventions are successful, there will still be a substantial number of people who will contract non-communicable diseases. The health burden of NCDs will grow for the foreseeable future; it will have a real impact on the health and non-health budgets of governments; it will have an impact on the GDP of countries; and it will have to be managed.

Without diminishing the primacy of prevention in global health, in this article we want to focus on the practicalities of the management of chronic diseases. Note again the shift from the NCDs vocabulary back to chronic diseases. This is intentional and pointed. If one is interested in understanding causes and prevention strategies it is important to separate the NCDs from other chronic diseases; however, if one is interested in the effects of the diseases, particularly on the health systems, then it is equally important to join the NCDs with other chronic diseases [9,14]. Many health conditions are chronic, and only some of those chronic health conditions are NCDs. Even after the inclusion – along with the core non-communicable diseases of cardiovascular disease, cancer, and diabetes – the respiratory conditions, mental health conditions, the arthritides, and functional loss and disability, there is a group of other diseases that are all chronic in nature. These are the communicable, infectious diseases that either have no cure, simply ongoing management (HIV/AIDS) or they have a cure, but the cure takes an extended period of 6 months treatment or more (tuberculosis and onchocerciasis – with some hope, following recent trials, that a shortened 2–week course may be feasible for tuberculosis treatment [20]). The commonality is chronicity – the temporal nature of the conditions requires an extended relationship with the health system, including quite probably an extended financial relationship.

Most low– and middle–income health systems have been designed for the management of maternal and neonatal mortality, and acute phases of infectious diseases such as malaria, respiratory tract infections, and diarrhoeal diseases [21]. “Receive them, Revive them, and Return them” could have been the motto emblazoned over the entrance gates to most health services in low and middle income countries. The system – beyond a record of immunisation or antenatal visits – has not traditionally needed to have a memory of the patient. For epidemiological purposes recording health systems interactions is important, but not central to the case management. For the acute diseases the diagnosis drives most of the decision process. In the management of chronic diseases, the diagnosis is known early in the patient–system relationship, and the ongoing strategy revolves around maintenance, monitoring, encouragement, and compliance (with acute services when necessary). This requires that a relationship is built with the client. However, a health system designed to deliver longitudinal management of a chronic health condition is distinctly different from one designed for the management of serial acute episodes.

The two main issues that arise when contemplating health systems' management of chronic diseases are structure and financing. Unfortunately, the research base for establishing evidence for action is thin. We return to the lack of research shortly. There is, however, little doubt about the financial impact of an increasing chronic disease burden on the individual, the family, and the health system. Under current health systems arrangements, the financing of chronic disease management in the population is costly, and at a national level costs will increase with rising prevalence [22]. One possibility is that the costs will be carried by individuals through out of pocket payments, which in low– and middle–income countries will often have catastrophic consequences for families [23]. Alternatively, costs could be carried by government, but few low– and middle–income countries could manage the entire financial burden, or some mixture of insurance, out of pocket payments, and government support.

With respect to the individual and family impact, quotes published in a recent article on catastrophic health care spending related to acute coronary syndrome in Kerala provide good examples [23]:

“I am not sure how long I can take my medicines. I have a credit account with the local pharmacy. They also help me out with samples from medical representatives. I cannot be a charity case forever, can I?”

and

“Right now, I am staying with one of my sisters, so that I don’t have to pay rent, water or electricity charges. My other sister has cut all ties with me. She fears that I will become a burden on her and her family.”

Both these quotes came from the same 50–year–old male patient and highlight individual and family collective financial burdens. The disease reduced his daily earning from US$ 17 per day to US$ 0.7 per day, and required an increase in expenditure to cover health care (although some was available through charity).

The impact on health systems, particularly health systems already stretched will be marked. In Kenya, the national government believes that the prevalence of type 2 diabetes in the population was around 10% in 2008, although “official statistics note a diabetes prevalence of 3.5%.” [24]. Under some fairly loose assumptions, one can imagine that in that 6.5% prevalence gap between what is believed and what is officially acknowledged, there is a fairly large group of people with insidious diabetes that is damaging their eyes, kidneys, and vascular system. For this chronic disease alone, the Kenyan government would be anticipating 10% of their population should be under clinical management (in 2008). Unlike treating a respiratory tract infection, the financing of diabetes management is a recurrent cost because of the chronic nature of the disease. Whence will that money come?

At the moment, 61% of the total health spending in Kenya goes to another chronic disease – HIV [25]. For that level of spending, antiretroviral coverage for 61% of HIV positive people in need of treatment has been achieved; meaning that 39% of people in need of treatment are missing out [26]. The commitment to provision of HIV treatment to those in need entails an expansion of services, and an increasing recurrent annual financial commitment that will not reduce in the near future. Indeed, given some of the evidence on antiretroviral resistance, one might imagine the cost will rather increase [27]. Furthermore, the more successful one becomes at management, the greater the number of people under management, the longer they will live, and the greater the recurrent costs.

**Figure:**
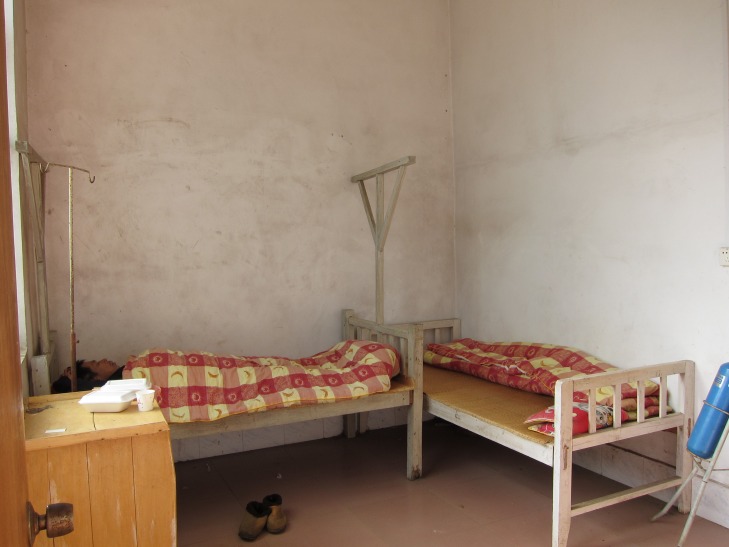
Photo: Courtesy of Dr Kit Yee Chan, personal collection

The purpose here is not to pit one disease against another and argue for the greater worthiness of one group of patients over another. The chronic communicable diseases and the chronic non-communicable diseases often have an interacting pathophysiology – and the management of one supports the management of the other. Both diabetes and HIV increase the likelihood of contracting TB [28]. Having diabetes increases the likelihood of chronic kidney disease, and chronic kidney disease increases the chance of heart failure [29]. Our interest is in the observation that the management of any chronic condition entails a commitment to recurrent costs, which reduces the flexibility of health systems to respond to new demands. It also requires that a health system that traditionally has a poor relationship with the population beyond acute management becomes more responsive to changes in the population health profiles. Such a system will be harder for poorer countries to manage that richer ones. The World Health Organization has suggested that [22]:

*“In order for low*– *and middle*–*income country health systems to expand individual health–care interventions [for chronic diseases], they need to prioritize a set of low*–*cost treatments that are feasible within their budgets. Many countries could afford a regimen of low–cost individual treatments by addressing inefficiencies in current operations for treating advanced*–*stage NCDs. Experiences from maternal and child health and infectious disease initiatives show that health priorities can be rearranged and low*–*cost individual treatments improved with only a modest injection of new resources*.”

Identifying inefficiencies and cost–effective interventions to improve health systems performance is laudable. Such a strategy will not, however, overcome the fundamental bottleneck. Health systems were never designed to treat 20% or more of a country's population as if they had a disease all the time. Take two middle–income countries as examples. In South Africa, the prevalence of HIV in adults is about 18% [30], diabetes is about 13% [31], and hypertension is 10% [32]. In Malaysia, the prevalence of diabetes in adults is about 15%, and 25% among one of the ethnic groups, the prevalence of hypertension is about 32% [33]. It is not enough to find cost–effective strategies for individual management. A fundamental rethink is required about how population health is managed when a substantial and growing proportion of the population has a chronic disease. We do not have the evidence base for that.

One approach to developing the evidence base is through “community health laboratories”. Essentially, within a tractable, geographically defined area, such as a county or district, health systems innovation can be tested and monitored [34]. Assuming that the entire population has been enumerated, and their health status and health systems interaction can be followed over time, it becomes possible to measure the impact of health systems innovations on various dimensions of health systems performance. Using these kinds of community settings, governments can look at implementation within the contexts of real lives and functioning communities. This is particularly important in environments where people employ pluralistic health care engaging multiple belief systems simultaneously, utilising both government and private providers. These community based research environments are particularly well suited to low– and middle–income countries.

In Malaysia, a new health and demographic surveillance site, the South East Asia Community Observatory (SEACO), is being established with the intention of being able to trial health systems innovation relevant to chronic disease management [35]. There are in excess of 40 health and demographic surveillance sites in the world, mainly located in low–income countries in sub–Saharan Africa [34]. They rely on enumerating and then following–up the population over time. The *raison d’être* of HDSS has been in the management and prevention of acute health conditions associated with vaccine trials, maternal and child health, malaria, diarrhoeal diseases, and HIV. Chronic diseases have emerged relatively recently within the scope of HDSS, and no sites had been established with this as a theme of interest. SEACO has been established with chronic diseases prevention and management as a central theme in its development. Unusually, it is also one of only two HDSS in middle–income countries.

The value of settings like SEACO is that they sit between the unrealistically controlled setting of an experimental trial – focused on the individual and uninterested in the contextual effects – and a completely realistic, unmonitored, community setting in which context is everything, but the impact of change cannot be measured or assessed. Low– and middle–income countries, faced with a growing chronic diseases problem will need to rethink how they deliver health care – and even what it may mean to deliver health care – but they also need an evidence base on which to make systems changes. The evidence generated through SEACO–like infrastructure has the potential to provide novel, yet realistic, models of prevention and health care management within the real life context of low– and middle–income countries. With the growing chronic diseases problem this evidence base and new ways of thinking are critical to making long term, sustainable systems change.
